# Supplementary material for: Disproportionality analysis of fondaparinux associated adverse events based on the FDA adverse event reporting system
Source: PLoS One. 2026 Feb 11;21(2):e0342548. doi: 10.1371/journal.pone.0342548 (PMC12893537; doi:10.1371/journal.pone.0342548)
Supplement: S1 File — (DOCX) [file pone.0342548.s001.docx]

**Supplementary 1:Contingency table and disproportionality analysis formulas**

**Tab. 1 Four grid table of adverse reaction signal of proportional imbalance method**

|  | fondaparinux-related ADEs | Non-drug-related ADEs | Total |
| --- | --- | --- | --- |
| fondaparinux | a | b | a + b |
| Non-drug | c | d | c + d |
| Total | a + c | b + d | N = a + b + c + d |

ADE, adverse drug events; a is the number of cases where a specific adverse event occurred after using fondaparinux; b is the number of cases where fondaparinux was used bu the specific adverse event did not occur, c is the number of cases where the specific adverse event occurred without he use of fondaparinux; d is the number of cases where neither fondaparinux was used nor the specific adverse event occurred.

**Tab. 2 Calculation formula and threshold**

| Method | Formula | ﻿Threshold |
| --- | --- | --- |
| ROR | $ROR=\frac{a / c}{b / d}$ | a ≥ 3  ROR ≥ 2  ﻿95%CI (lower limit) > 1 |
|  | $SE(lnROR)=\sqrt{\frac{1}{a}+\frac{1}{b}+\frac{1}{c}+\frac{1}{d}}$ |  |
|  | $95\%CI= e^{\ln\left( ROR \right)\pm1.96se}$ |  |
| PRR | $PRR=\frac{a / (a+b)}{c / (c+d)}$ | a ≥ 3  PRR ≥ 2  ﻿95%CI (lower limit) > 1 |
|  | $SE(lnPRR)=\sqrt{\frac{1}{a}-\frac{1}{a+b}+\frac{1}{c}-\frac{1}{c+d}}$ |  |
|  | $95\%CI= e^{\ln\left( PRR \right)\pm1.96se}$ |  |
| BCPNN | $IC=\log_{2}\frac{p(x, y)}{p\left( x \right)p(y)}= {log}_{2}\frac{a(a+b+c+d)}{(a+b)(a+c)}$ | IC025>0 |
|  | $E(IC)=\log_{2}\frac{(a+\gamma ij)(a+b+c+d+\alpha)(a+b+c+d+\beta)}{\left( a+b+c+d+\gamma\right)(a+b+\alpha i)(a+c+\beta j)}$ |  |
|  | $V\left( \mathrm{IC} \right)=\frac{1}{{(ln2)}^{2}}[\frac{\left( a+b+c+d \right)-a+\gamma-\gamma ij}{\left( a+\gamma ij \right)\left( 1+a+b+c+d+\gamma\right)}+\frac{\left( a+b+c+d \right)-\left( a+b \right)+a-\alpha1}{\left( a+b+\alpha i \right)\left( 1+a+b+c+d+\alpha\right)}+\frac{\left( a+b+c+d+\alpha\right)-\left( a+c \right)+\beta-\beta j}{\left( a+b+\beta j \right)\left( 1+a+b+c+d+\beta\right)}]$ |  |
|  | $\gamma=\gamma\mathrm{ij}\frac{(a+b+c+d+\alpha)(a+b+c+d+\beta)}{\left( a+b+\alpha i \right)(a+c+\beta j)}$ |  |
|  | $\mathrm{IC}025=E\left( \mathrm{IC} \right)-2 \sqrt{V(IC)}$ |  |
| EBGM | $EBGM=\frac{a(a+b+c+d)}{\left( a+c \right)(a+b)}$ | EBGM05>2 |
|  | $SE(lnEBGM)=\sqrt{\frac{1}{a}+\frac{1}{b}+\frac{1}{c}+\frac{1}{d}}$ |  |
|  | $95\%CI= e^{\ln\left( EBGM \right)\pm1.96se}$ |  |

N,the number of reports; a is the number of cases where a specific adverse event occurred after using Fondaparinux; b is the number of cases where Fondaparinux was used but the specific adverse event did not occur; c is the number of cases where the specific adverse event occurred without the use of Fondaparinux,d is the number of cases where neither Fondaparinux was used nor the specific adverse event occurred; ROR, reporting odds ratio; $\gamma$,$\gamma i$j represent the parameters of the Dirichlet distribution; a, a$i$,$\beta j$, represent the parameters of the Beta distribution; BCPNN, bayesian confidence propagation neural network, PRR, proportional reporting ratio; EBGM, empirical bayes geometric mean; IC025, the lower limit of 95% Cl for the IC; E(IC),the IC, expectations; V(IC),the variance of IC; EBGM05, the lower limit of the 95% Cl, for EBGM.
