# Supplementary material for: Disproportionality analysis of fondaparinux associated adverse events based on the FDA adverse event reporting system
Source: PLoS One. 2026 Feb 11;21(2):e0342548. doi: 10.1371/journal.pone.0342548 (PMC12893537; doi:10.1371/journal.pone.0342548)
Supplement: S3 File — (DOCX) [file pone.0342548.s003.docx]

Supplementary Table 3. Disproportionality analysis of major bleeding- and HIT-related adverse events across different anticoagulant agents in the FAERS database

| Drug | PT | number | ROR(95%Cl) | PRR(chisq) | EBGM(EBGM05) | IC(IC025) |
| --- | --- | --- | --- | --- | --- | --- |
| Enoxaparin | Haemorrhage | 884 | 17 ( 15.89 - 18.18 ) | 16.56 ( 12820.83 ) | 16.41 ( 15.34 ) | 4.04 ( 3.91 ) |
| Enoxaparin | Haematoma | 466 | 33.82 ( 30.84 - 37.09 ) | 33.35 ( 14341.17 ) | 32.71 ( 29.83 ) | 5.03 ( 4.8 ) |
| Enoxaparin | Muscle Haemorrhage | 136 | 69.59 ( 58.6 - 82.64 ) | 69.31 ( 8788.61 ) | 66.56 ( 56.05 ) | 6.06 ( 5.24 ) |
| Enoxaparin | Heparin-Induced Thrombocytopenia | 313 | 125 ( 111.38 - 140.28 ) | 123.8 ( 35480.76 ) | 115.27 ( 102.71 ) | 6.85 ( 6.23 ) |
| Unfractionated Heparin | Haemorrhage | 456 | 7.89 ( 7.2 - 8.66 ) | 7.8 ( 2696.1 ) | 7.77 ( 7.08 ) | 2.96 ( 2.8 ) |
| Unfractionated Heparin | Haematoma | 199 | 12.99 ( 11.29 - 14.94 ) | 12.92 ( 2171.74 ) | 12.82 ( 11.15 ) | 3.68 ( 3.39 ) |
| Unfractionated Heparin | Muscle Haemorrhage | 26 | 11.77 ( 8 - 17.32 ) | 11.76 ( 254.13 ) | 11.68 ( 7.94 ) | 3.55 ( 2.51 ) |
| Unfractionated Heparin | Heparin-Induced Thrombocytopenia | 2347 | 1772.87 ( 1670.55 - 1881.45 ) | 1655.66 ( 1858862.99 ) | 793.43 ( 747.64 ) | 9.63 ( 9.14 ) |
| Apixaban | Haemorrhage | 3937 | 9.82 ( 9.5 - 10.14 ) | 9.68 ( 29339.57 ) | 9.3 ( 9 ) | 3.22 ( 3.17 ) |
| Apixaban | Haematoma | 977 | 9.09 ( 8.52 - 9.69 ) | 9.06 ( 6716.97 ) | 8.72 ( 8.18 ) | 3.13 ( 3.02 ) |
| Apixaban | Muscle Haemorrhage | 96 | 6.12 ( 5 - 7.5 ) | 6.12 ( 399.81 ) | 5.98 ( 4.88 ) | 2.58 ( 2.21 ) |
| Apixaban | Heparin-Induced Thrombocytopenia | 14 | 0.65 ( 0.39 - 1.11 ) | 0.65 ( 2.54 ) | 0.66 ( 0.39 ) | -0.61 ( -1.32 ) |
| Dabigatran | Haemorrhage | 2871 | 12.19 ( 11.74 - 12.66 ) | 11.98 ( 28000.59 ) | 11.62 ( 11.2 ) | 3.54 ( 3.48 ) |
| Dabigatran | Haematoma | 601 | 9.45 ( 8.71 - 10.25 ) | 9.42 ( 4407.71 ) | 9.2 ( 8.48 ) | 3.2 ( 3.06 ) |
| Dabigatran | Muscle Haemorrhage | 37 | 3.98 ( 2.88 - 5.51 ) | 3.98 ( 81.74 ) | 3.95 ( 2.86 ) | 1.98 ( 1.4 ) |
| Dabigatran | Heparin-Induced Thrombocytopenia | 8 | 0.64 ( 0.32 - 1.28 ) | 0.64 ( 1.59 ) | 0.64 ( 0.32 ) | -0.64 ( -1.54 ) |
| Edoxaban | Haemorrhage | 88 | 9.63 ( 7.8 - 11.89 ) | 9.5 ( 669.44 ) | 9.49 ( 7.69 ) | 3.25 ( 2.81 ) |
| Edoxaban | Haematoma | 17 | 6.95 ( 4.32 - 11.2 ) | 6.94 ( 86.34 ) | 6.93 ( 4.31 ) | 2.79 ( 1.7 ) |
| Edoxaban | Muscle Haemorrhage | 1 | 2.84 ( 0.4 - 20.2 ) | 2.84 ( 1.19 ) | 2.84 ( 0.4 ) | 1.51 ( -1.48 ) |
| Rivaroxaban | Haemorrhage | 6291 | 15.78 ( 15.38 - 16.2 ) | 15.43 ( 79062.56 ) | 14.42 ( 14.05 ) | 3.85 ( 3.81 ) |
| Rivaroxaban | Haematoma | 1743 | 16.33 ( 15.55 - 17.15 ) | 16.23 ( 23079.51 ) | 15.1 ( 14.38 ) | 3.92 ( 3.83 ) |
| Rivaroxaban | Muscle Haemorrhage | 242 | 15.68 ( 13.76 - 17.87 ) | 15.67 ( 3086.3 ) | 14.62 ( 12.83 ) | 3.87 ( 3.6 ) |
| Rivaroxaban | Heparin-Induced Thrombocytopenia | 23 | 1.05 ( 0.69 - 1.58 ) | 1.05 ( 0.05 ) | 1.05 ( 0.69 ) | 0.06 ( -0.53 ) |
