# Supplementary material for: Disproportionality analysis of fondaparinux associated adverse events based on the FDA adverse event reporting system
Source: PLoS One. 2026 Feb 11;21(2):e0342548. doi: 10.1371/journal.pone.0342548 (PMC12893537; doi:10.1371/journal.pone.0342548)
Supplement: Attachment — Submitted filename: Response to Reviewers.docx [file pone.0342548.s008.docx]

Dear Dr. Genn Givero,

Thank you very much for your email and the constructive comments regarding my manuscript titled “Disproportionality Analysis of Adverse Drug Events Related to Fondaparinux: Based on the FDA Adverse Event Reporting System (FAERS) Database” (Manuscript ID: PONE-D-25-40001).

I have carefully revised the manuscript to address the concerns about text overlap. The duplicated parts have been thoroughly rephrased, and all relevant sources are now properly cited. To comply with the requirements, some figures and tables containing formulas that could not be further modified to reduce similarity have been moved to the supplementary materials.

Additionally, I have attached the similarity (plagiarism) report for your reference.

The revised manuscript, supplementary files, and similarity report have been uploaded to the Editorial Manager system for your consideration. Please kindly let me know if you require any further information or additional modifications.

Thank you again for your valuable feedback and for the opportunity to improve the manuscript.

Best regards,

Yuan Bian
