# Supplementary material for: Disproportionality analysis of fondaparinux associated adverse events based on the FDA adverse event reporting system
Source: PLoS One. 2026 Feb 11;21(2):e0342548. doi: 10.1371/journal.pone.0342548 (PMC12893537; doi:10.1371/journal.pone.0342548)
Supplement: Attachment — Submitted filename: Review Article.docx [file pone.0342548.s007.docx]

**Review report — *Disproportionality Analysis of Adverse Drug Events Related to Fondaparinux: Based on the FAERS Database***

Hematoma/hemorrhage/hematoma are expected in surgical patients receiving anticoagulants. The FAERS analysis must try to separate background risk (e.g., post-operative bleeding) from drug-specific signal.

At minimum, report how many bleeding/hematoma reports included concomitant heparin/LMWH or warfarin or antiplatelet agents. How many HIT-like and TTS reports had recent/ concurrent heparin exposure? Provide stratified results.

For the HIT-like signals and thrombosis-with-thrombocytopenia, present how many reports had recent heparin exposure or concurrent heparin/LMWH. Without this, attributing HIT to fondaparinux is prematureWhile the study identifies specific adverse events, the small number of reports for certain signals (e.g., abnormal concentrations of coagulation factor X) raises concerns regarding the statistical power and clinical significance of these findings.

The retrospective nature of the analysis does not allow for establishing causality; only associations can be suggested. This aspect significantly limits the implications of the findings in clinical practice.

Absence of detailed laboratory data (e.g., anti-PF4 antibody quantification) diminishes the ability to explore the mechanisms underlying the reported adverse events. Including such data could strengthen the conclusions.

The authors should provide a more detailed discussion on the limitations of using FAERS and the implications of biases stemming from spontaneous reporting.

If Excel was used only for tabulation/plotting, state that explicitly and explain how reproducible calculations (ROR, PRR, BCPNN, EBGM) were done and by which package/tool.

Some years/statements may need cited support (for example, the statement about Fondaparinux being contraindicated in the US/Canada for CrCl <30 mL/min — cite the product monograph).

Compute disproportionality of bleeding/HIT signals for fondaparinux *vs* enoxaparin, unfractionated heparin, and DOACs. This helps determine whether signals are drug-specific or class effects.There is a need to develop concrete clinical recommendations based on findings, particularly for monitoring patients, especially vulnerable populations such as the elderly.

Define all abbreviations on first use (e.g., AE, PT, SOC, TTO, HIT, VITT).

Most reports are from Europe and the United States, leading to questions about the applicability of findings in other populations, particularly in Asia and other regions with different healthcare practices.

The authors should consider proposing methods to collect data from underrepresented regions to ensure that findings are applicable globally.
