# Supplementary material for: Disproportionality analysis of fondaparinux associated adverse events based on the FDA adverse event reporting system
Source: PLoS One. 2026 Feb 11;21(2):e0342548. doi: 10.1371/journal.pone.0342548 (PMC12893537; doi:10.1371/journal.pone.0342548)
Supplement: Attachment — Submitted filename: Response_to_Reviewers_auresp_1.docx [file pone.0342548.s009.docx]

Letter to the Editor and Reviewers

Dear Editor and Reviewers,

We would like to express our sincere gratitude for your careful review and constructive feedback on our manuscript. We truly appreciate the time and effort that the editor and the three reviewers have devoted to providing detailed and insightful comments, which have been instrumental in improving the scientific rigor, logical clarity, and international quality of our work.

After receiving the comments, we thoroughly analyzed and addressed each point. The revised manuscript has been substantially improved in research design, data interpretation, and academic presentation. The main revisions and improvements are summarized as follows:

Title and Abstract

The title has been refined for academic precision. All abbreviations (FAERS, ROR, PRR, BCPNN, EBGM) are now defined upon first use in both the Abstract and the main text to enhance clarity.

Methodological Transparency

The FAERS database citation was added, and detailed descriptions of data extraction and cleaning were included. The R packages (with version numbers) used for disproportionality analysis are now specified. Section 2.3 clarifies the rationale for signal detection thresholds with appropriate literature support, ensuring reproducibility and compliance with CIOMS VIII guidelines.

Results and Stratified Analyses

The results section was reorganized for logical consistency. Additional stratified analyses were conducted for reports involving concomitant antiplatelet or anticoagulant exposure to address concerns regarding background surgical bleeding risk.

Discussion Revision

The discussion was extensively restructured to distinguish established anticoagulant related adverse events (e.g., hemorrhage, hematoma) from exploratory or hypothesis generating signals (e.g., HIT like reactions, abnormal factor X concentrations). Overinterpretations were removed, and clearer explanations were added regarding the limitations of FAERS based inference.

Expanded Limitations Section

A dedicated subsection has been expanded to emphasize FAERS limitations, including underreporting, duplication, missing covariates, lack of exposure denominators, reporting bias, and geographic imbalance (predominantly U.S. and European data). Approximately 30% of reports lacked outcome data, which is now explicitly highlighted.

Figures, Tables, and Language Editing

Figure legends and table captions were revised to be self explanatory. Table formats were standardized, and the incorrect drug name in Figure 2 was corrected. The generic name “fondaparinux” is used consistently, and all grammatical and stylistic issues were carefully revised to improve readability.

Ethical Statement and Conclusion

An ethical statement has been added, clarifying that ethical approval was not required because FAERS is a publicly available, de-identified database. The conclusion was rewritten to remove unsupported recommendations, ensuring that the findings are presented cautiously and empirically.

Funder and Competing Interests

We have updated both the Role of Funder and Competing Interests statements as requested, including the required sentence in full.

We believe that these substantial revisions have greatly enhanced the scientific quality, transparency, and overall coherence of the paper. We sincerely thank the editor and reviewers for their detailed and constructive comments, which have guided us in refining the manuscript to its current improved version.

Editor comment 1: Methodological Clarity and Reproducibility

Issue: The methods section lacks clarity on specific R scripts or packages used for data extraction, cleaning, and analysis (e.g., version numbers for openFDA API, R packages for BCPNN/EBGM computation).

Response:

We greatly appreciate the editor’s constructive comment. In the revised manuscript, we have substantially enhanced the transparency and reproducibility of the analytical methods.

Sections 2.2 through 2.6 now provide a detailed description of the entire workflow, including data extraction, cleaning, MedDRA coding, and signal detection. The R version (4.3.0) and all major packages used (dplyr, data.table, epiR, DescTools, bayesAB, openEBGM, and ggplot2) are explicitly listed. The threshold criteria for each algorithm are specified as follows: ROR (lower 95% CI > 1), PRR (≥ 2 with χ² ≥ 4), BCPNN (IC025 > 0), and EBGM (EB05 > 1).

We also clarified that all analyses were executed using fully scripted workflows in R Studio and cross-verified in Microsoft Excel for accuracy. These revisions align with the CIOMS VIII recommendations and ensure full methodological transparency and reproducibility of our study.

Editor comment 2: Lack of Validation Against Comparator Drugs or Negative Controls

Issue: The study focuses solely on fondaparinux without contextual comparison to similar anticoagulants (e.g., enoxaparin, dalteparin, rivaroxaban), limiting interpretability of the disproportionality signals.

Response:

We thank the editor for this valuable and insightful recommendation. In the revised manuscript, we have added and expanded comparative and sensitivity analyses, as presented in Section 3.2.3 “Stratified and comparative analysis across anticoagulant exposures.”

First, a stratified analysis was performed to compare the disproportionality of major adverse events in fondaparinux users with and without concomitant anticoagulant or antiplatelet therapy. The significant bleeding signals (hematoma, muscle haemorrhage, and haemorrhage) persisted even after excluding concomitant medications, indicating that these associations were largely independent of polypharmacy.

Second, a comparative disproportionality analysis was conducted across other major anticoagulant classes, including enoxaparin, unfractionated heparin (UFH), and direct oral anticoagulants (DOACs: apixaban, dabigatran, edoxaban, and rivaroxaban), using identical algorithms (ROR, PRR, BCPNN, EBGM) and detection thresholds. The results showed consistent positive bleeding signals across all anticoagulants, confirming a class- wide effect, whereas HIT related signals were observed only for fondaparinux and UFH, suggesting drug specific associations.

These additions have been incorporated into both the Results and Discussion sections, supported by relevant literature and regulatory references, thereby strengthening the interpretability and robustness of our findings.

Editor comment 3: Overinterpretation of Signal Strength (Causality vs. Association)

Issue: The Discussion implies causal relationships (e.g., “fondaparinux may induce HIT” or label change suggestions), which exceeds what FAERS data can support.

Response:

We sincerely appreciate the editor’s valuable observation. The Discussion and Conclusion have been thoroughly reviewed and revised to avoid implying causality:

All potentially causal expressions were rewritten into statistical association or signal based language—for example, “may induce” was replaced with “was associated with HIT like reactions,” and these were explicitly defined as case report level and hypothesis generating findings;

Sentences suggesting label changes or recommendations beyond the strength of evidence were removed;

We added explicit statements at the beginning of Section 4 (Discussion) and in Section 4.5 (Limitations) clarifying that FAERS is intended for disproportionality signal detection and hypothesis generation, not causal inference;

Phrases that might imply causality (e.g., “likely pharmacologically driven,” “underscore the importance of… monitoring”) were replaced with more cautious wording such as “may reflect pharmacologic plausibility” and “support prioritizing monitoring as an exploratory signal”;

In the TTO (Section 4.4) and Rare events (Section 4.3) subsections, we included explicit cautionary statements such as “should be interpreted cautiously” and “should be regarded as exploratory and hypothesis generating,” emphasizing that these observations are exploratory and warrant further validation.

These revisions ensure that all interpretations are scientifically cautious, aligned with the evidentiary boundaries of FAERS data, and consistent across the manuscript.

Editor comment 4: Limited Consideration of Reporting Bias and Confounding

Issue: Although reporting bias is briefly mentioned, the manuscript does not adequately address consumer reporting proportion, indication confounding, or the lack of exposure denominators.

Response:

We appreciate the editor’s valuable feedback. The Limitations section (Section 4.5) has been expanded to provide a more thorough discussion of potential reporting bias and confounding factors. The key additions include:

Reporting source imbalance: Approximately 45.91% of reports were submitted by consumers, which may affect data accuracy and clinical interpretation;

Indication confounding and background risk: Because fondaparinux is frequently administered for postoperative thromboprophylaxis, bleeding events may be partly attributable to surgical factors rather than drug specific effects. We have added a statement acknowledging that residual confounding may remain even after excluding concomitant anticoagulants or antiplatelet agents;

Lack of exposure denominators: We noted that FAERS lacks patient level exposure data, making it impossible to calculate absolute incidence or risk; the present findings therefore represent disproportionality signals rather than measures of relative risk;

Geographic imbalance and external validation: The dataset is dominated by U.S. and European reports, with minimal Asian data. We recommend future studies to triangulate findings using multiple pharmacovigilance databases such as VigiBase and EudraVigilance to enhance external validity.

These revisions provide a more comprehensive and balanced acknowledgment of potential reporting bias and confounding within the study limitations.

Editor comment 5: Data Presentation and Consistency Issues

Issue: Figure 2 caption incorrectly refers to “Ambrisentan” instead of “Fondaparinux.” Inconsistent capitalization and redundant labeling also appear in tables and figures.

Response:

We sincerely thank the editor for this meticulous observation. All figures and tables have been carefully proofread and revised for consistency:

Correction of figure titles: The caption of Figure 2 has been corrected from “Ambrisentan” to “Fondaparinux,” and all figure titles have been verified for accuracy;

Terminology and formatting standardization: Abbreviations such as SOC (System Organ Class), PT (Preferred Term), and TTO (Time to Onset) have been consistently defined and explained within the figure legends;

Improved self explanatory legends: Each figure and table (Figures 1–5, Tables 1–4) now includes a complete and standalone legend, ensuring they can be interpreted independently of the main text;

Text–figure alignment: Cross references in the text (e.g., “Figure 3 visualizes …,” “Figure 4 illustrates …”) were checked for full correspondence with figure content.

These revisions enhance the accuracy, readability, and overall presentation quality of the manuscript.

Editor comment 6: Clinical Interpretation of Rare Events

Issue: The discussion highlights rare events such as abnormal coagulation factor X concentration (n = 3) and antiphospholipid syndrome (n = 6) as potential novel signals but lacks adequate caution regarding the reliability of such small counts.

Response:

We thank the editor for this thoughtful and important comment. The relevant text in Section 4.3 “Rare reports” has been carefully revised to ensure cautious interpretation of rare signals.

All findings based on very limited numbers of cases (e.g., abnormal coagulation factor X concentration, thrombosis with thrombocytopenia, antiphospholipid syndrome, eosinophilia, thrombocytosis) are now explicitly described as exploratory or hypothesis generating.

We added qualifying statements such as “should be interpreted cautiously” and “should be regarded as exploratory and hypothesis generating” to emphasize that these results are preliminary and require further validation.

These revisions ensure that the discussion accurately reflects the statistical and exploratory nature of FAERS data while maintaining scientific caution.

Editor comment 7: Incomplete Integration with Existing Literature and Regulatory Implications

Issue: The discussion lacks engagement with prior pharmacovigilance studies and regulatory updates from FDA or EMA, limiting the contextual and regulatory interpretation of the findings.

Response:

We appreciate the editor’s valuable recommendation. In the revised manuscript, we have strengthened the integration with existing literature and regulatory information, particularly in Section 4.2 and the closing paragraphs of the Discussion.

Comparison with prior studies: Additional discussion was added comparing our findings with previous pharmacovigilance analyses (e.g., Frontiers in Oncology, 2023), showing that the identified hematologic and bleeding signals for fondaparinux are consistent with earlier postmarketing observations;

Inclusion of regulatory information: References to the 2024 FDA prescribing information and the 2024 EMA EPAR (Product Information for Arixtra) were incorporated to highlight that both agencies list haemorrhage and thrombocytopenia in fondaparinux’s safety labeling and recommend renal function monitoring;

Class level context: We noted the alignment between fondaparinux and other factor Xa inhibitors in terms of safety profile and regulatory labeling, reinforcing the external validity of our findings;

Avoidance of overinterpretation: Statements proposing label modification or clinical changes were removed, ensuring that our conclusions focus on pharmacovigilance observation rather than regulatory action.

These revisions enhance the manuscript’s scholarly depth, align it with current regulatory knowledge, and strengthen the contextual interpretation of the findings.

Reviewer 1 – Comment 1

Comment:

The Abstract contains many abbreviations that are not defined (e.g., FAERS, ROR, PRR, BCPNN, EBGM). Ideally these should be defined with first use in both the Abstract and main text.

Response:

We thank the reviewer for this helpful suggestion. In the revised manuscript, all abbreviations have been clearly defined at their first appearance in both the Abstract and the main text. Specifically, the following definitions were added: FAERS = FDA Adverse Event Reporting System; ROR = Reporting Odds Ratio; PRR = Proportional Reporting Ratio; BCPNN = Bayesian Confidence Propagation Neural Network; and EBGM = Empirical Bayes Geometric Mean. These clarifications improve the readability and precision of the manuscript.

Reviewer 1 – Comment 2

Comment:

Figure 2 – The incorrect drug (ambrisentan) is listed in the figure title. Please ensure that the data apply to fondaparinux and not to ambrisentan.

Response:

We appreciate the reviewer’s careful observation. All data have been verified to correspond exclusively to fondaparinux. The caption of Figure 2 has been corrected from “Ambrisentan” to “Fondaparinux,” and the figure legend and corresponding text references have been updated accordingly.

Reviewer 1 – Comment 3

Comment:

Fondaparinux is capitalized throughout the manuscript. It should not be capitalized as it is a generic name.

Response:

Thank you for noting this. All occurrences of “Fondaparinux” have been changed to lowercase “fondaparinux,” and capitalization has been standardized for all generic drug names throughout the manuscript.

Reviewer 1 – Comment 4

Comment:

Table 1 – For age, please specify what is being shown? Is it a mean or median? Is the range an IQR? Please also define “tto” and “ttoQ” in the table or table legend.

Response:

We appreciate this helpful comment. In the revised Table 1, the data presentation has been clarified. Continuous variables such as Age (years) and Time to onset (days) are now explicitly expressed as median (interquartile range, IQR), as stated in the table footnote. The previous abbreviations “TTO” and “TTOQ” have been removed to improve readability and ensure consistency across the manuscript.

Reviewer 1 — Comment 5

Comment ：

“The authors write, ‘…most adverse events in both male and female patients occurred within the first month of treatment…’ Would it be more accurate to say, ‘…within the first month of INITIATING treatment…’?”

Response:

We thank the reviewer for this valuable suggestion. We agree that the original wording could cause ambiguity regarding the timing of adverse event onset. The sentence has been revised as follows to improve clarity:

“Figure 5 illustrates that most adverse events in both male and female patients occurred within the first month of initiating treatment.”

This revision clarifies the temporal meaning and avoids potential misunderstanding.

Reviewer 1 — Comment 6

The authors write, “However, it remains necessary to continue safety monitoring throughout the entire treatment period and during the extended follow-up phase, potentially up to one year after drug discontinuation.”

The reviewer disagrees, noting that with a half-life of ~18 hours, it is unrealistic that fondaparinux would contribute to adverse events long after discontinuation.

Response:

We appreciate the reviewer’s insightful comment. We agree that the original statement was not pharmacologically justified given the approximately 18 hour half-life of fondaparinux. The sentence has been revised to reflect a more appropriate interpretation:

“Although a small number of events were reported beyond the initial treatment period, these may reflect delayed reporting rather than delayed drug effects. Therefore, it remains important to maintain vigilance for adverse events during the active treatment phase and shortly after drug discontinuation.”

This revision aligns the discussion with the pharmacokinetic properties of fondaparinux and avoids overstating the duration of risk after discontinuation.

Reviewer 1 – Comment 7

Comment:

It is remarkable that AE reporting for fondaparinux has significantly decreased over the last several years. It would be interesting if the authors speculated as to why this might be. Is it because the drug is no longer new and there is thus less incentive to report adverse effects? Or could it be because it is used far less than it once was, possibly due to increased use of direct oral anticoagulants?

Response:

We thank the reviewer for this thoughtful suggestion. The Discussion has been expanded to explain the declining trend in fondaparinux adverse event reports. The revised text notes that the overall reduction in AE reporting after 2015 likely results from two concurrent factors:

(1) a progressive decrease in clinical utilization of fondaparinux, as direct oral anticoagulants (DOACs) have become the preferred anticoagulants for many thromboembolic indications; and

(2) a decline in spontaneous reporting once a drug is well established and its safety profile is familiar to healthcare professionals, reducing motivation to submit new reports.

The relevant paragraph in the Discussion now reads:

“The marked decline in fondaparinux related AE reporting after 2015 likely reflects two concurrent trends. First, clinical utilization of fondaparinux has progressively decreased as DOACs became the preferred anticoagulants in many thromboembolic indications. Second, spontaneous reporting typically diminishes once a drug becomes well established and its safety characteristics are familiar to prescribers, reducing the motivation to report.”

This revision provides a rational explanation for the temporal reporting pattern and aligns the interpretation with real-world pharmacovigilance behavior.

Reviewer 1 – Comment 8

Comment:

It is worth noting that hematologists do not recognize a single disorder called “thrombosis and thrombocytopenia syndrome”. Rather, this is a group of disorders associated with both thrombosis and thrombocytopenia including but not limited to heparininduced thrombocytopenia, other PF4 disorders such as vaccineinduced thrombotic thrombocytopenia, antiphospholipid syndrome, disseminated intravascular coagulation, and thrombotic thrombocytopenic purpura.

Response:

Thank you for this important clarification. We agree that “thrombosis with thrombocytopenia syndrome (TTS)” is not a single clinical disorder but a descriptive grouping used in pharmacovigilance databases. In our analysis, this term reflects the standardized MedDRA Preferred Term applied within FAERS for coding purposes rather than a unified hematologic diagnosis.

To address the reviewer’s concern, we have revised the manuscript to clarify the nature of this PT, emphasized that such reports may correspond to various underlying conditions (e.g., HIT, VITT, PF4 related immune thrombosis, or other thrombotic–thrombocytopenic disorders), and avoided implying that TTS represents a distinct clinical entity. We have also added a general explanatory statement in the Methods to ensure consistent and accurate interpretation of MedDRA terminology throughout the manuscript.

These revisions ensure that the terminology is accurate, clinically appropriate, and aligned with hematologic expertise.

Reviewer 2 – Comment 1

Comment:

Title of the study should be revised.

Response:

We appreciate the reviewer’s suggestion. The title has been revised to more accurately reflect the study design, data source, and analytical approach. The current title now reads:

“Disproportionality Analysis of Fondaparinux Associated Adverse Events Based on the FDA Adverse Event Reporting System (FAERS)”

This revised title explicitly indicates that the study employed a disproportionality analysis using FAERS data, thereby improving scientific clarity and alignment with pharmacovigilance research conventions.

Reviewer 2 – Comment 2

Comment:

Introduction is a bit short and avoid redundancy, revise accordingly.

Response:

Thank you for this helpful comment. We carefully reviewed the Introduction and made targeted revisions to improve its clarity, structure, and coherence. While the overall length was not substantially increased, several important improvements were made:

Redundant descriptions were removed to avoid repetition and improve readability.

Background information on fondaparinux and its clinical use was streamlined and presented in a more concise and logical manner.

The rationale for conducting a post-marketing pharmacovigilance study using FAERS was clarified to better connect the background with the study objectives.

Transitions between paragraphs were refined to ensure a smoother flow of ideas.

These revisions enhance the clarity and focus of the Introduction while maintaining concise presentation.

Reviewer 2 – Comment 3

Comment:

The manuscript often implies causal links, but FAERS can only generate signals, not establish causality. Stronger disclaimers are needed throughout; revise accordingly for better clarity and impact.

Response:

We thank the reviewer for highlighting this important point. In response, we have carefully reviewed the entire manuscript and revised any expressions that could be interpreted as implying causality. Throughout the Introduction, Results, Discussion, and Conclusion, we now explicitly emphasize that:

FAERS is a spontaneous reporting system (SRS) and cannot establish causal relationships.

All disproportionality metrics (ROR, PRR, BCPNN, EBGM) indicate statistical associations, not risk estimates.

The findings should be interpreted as hypothesi generating signals that require validation in epidemiologic or mechanistic studies.

Examples of revisions include:

In the Introduction, we added:

“Given the inherent limitations of spontaneous reporting systems, these findings should be interpreted as hypothesis generating rather than causal.”

In the Discussion, we strengthened the disclaimer by stating:

“The FAERS database allows detection of disproportionate reporting but does not establish causal relationships between fondaparinux and adverse events.”

In the Conclusion, we revised the wording to ensure that the study “identifies safety signals” rather than implying causal effects or clinical recommendations.

Together, these modifications ensure that the manuscript accurately reflects the methodological limitations of FAERS and avoids overstating causality.

Reviewer 2 – Comment 4

Comment:

FAERS reports are prone to underreporting, duplication, missing covariates, and confounding. Although limitations are mentioned, their implications for interpretation are underplayed. Discussion all the issues accordingly throughout the revised manuscript.

Response:

We thank the reviewer for this important methodological comment. In the revised manuscript, we have substantially expanded the Limitations section and strengthened the discussion of how FAERS related biases impact interpretation. Specifically, we added or emphasized the following points:

Underreporting and selective reporting may distort the observed distribution of adverse events and lead to underestimation of clinically relevant outcomes.

Duplicate reporting remains an inherent issue despite our de-duplication procedure, and residual duplication may still inflate signal strength.

Missing clinical covariates (e.g., comorbidities, indications, laboratory values, dosage, duration of therapy) limit the ability to adjust for confounding.

Confounding by indication and procedural risk, especially in surgical patients receiving fondaparinux, complicates interpretation of bleeding and thrombocytopenia signals.

Lack of exposure denominators prevents estimation of incidence or absolute risk.

Reporting heterogeneity (e.g., high proportion of consumer reports, geographic imbalances) may influence signal patterns.

Stimulated reporting associated with label changes or new indications was addressed as a potential source of temporal variation.

These additions have been incorporated into the revised Discussion (Section 4.5) to highlight how these limitations constrain causal interpretation and generalizability.

Together, these revisions provide a more balanced, transparent, and rigorous interpretation of the findings.

Reviewer 2 – Comment 5

Comment:

Cite reference for FAERS in method part.

Response:

Thank you for this suggestion. We have added an appropriate citation for the FAERS database in the Methods (Section 2.1) to ensure proper attribution. The revised text includes the following reference:

1. van Puijenbroek EP, Bate A, Leufkens HGM, Lindquist M, Orre R, Egberts ACG. A comparison of measures of disproportionality for signal detection in spontaneous reporting systems for adverse drug reactions. Pharmacoepidemiol Drug Saf. 2002 Jan–Feb;11(1):3–10. doi: 10.1002/pds.668.
2. Evans SJ, Waller PC, Davis S. Use of proportional reporting ratios (PRRs) for signal generation from spontaneous adverse drug reaction reports. Pharmacoepidemiol Drug Saf. 2001 Oct–Nov;10(6):483–486. doi: 10.1002/pds.677.
3. 3.Bate A, Lindquist M, Edwards IR, Olsson S, Orre R, Lansner A, De Freitas RM. A Bayesian neural network method for adverse drug reaction signal generation. Eur J Clin Pharmacol. 1998 Jun;54(4):315–321. doi: 10.1007/s002280050466.

4.DuMouchel W. Bayesian data mining in large frequency tables, with an application to the FDA Spontaneous Reporting System. Am Stat. 1999 Aug;53(3):177–190. doi: 10.2307/2686093.

5.Bate A, Evans SJW. Quantitative signal detection using spontaneous ADR reporting. Pharmacoepidemiol Drug Saf. 2009 Jun;18(6):427–436. doi: 10.1002/pds.1742

This citation is now included in the reference list and formatted according to journal requirements

.Reviewer 2 – Comment 6

Comment:

Also cite relevant references in the method part i.e. Medex_UIMA_1.8.3 system, Preferred Terms (PTs) and System Organ Classes (SOCs) from MedDRA, Reporting Odds Ratio (ROR), Proportional Reporting Ratio (PRR), Bayesian Confidence Propagation Neural Network (BCPNN), and Empirical Bayesian Geometric Mean (EBGM).

Response:

Thank you for this helpful suggestion. In the revised manuscript, we have added complete and formally cited references for all tools, terminologies, and disproportionality algorithms used in the study. Specifically:

For MedEx_UIMA 1.8.3, the reference “MedEx: a medication information extraction system for clinical narratives (10.1197/jamia.M3378)” has been added.

For MedDRA (PTs and SOCs), the reference “Using MedDRA: implications for risk management (DOI: 10.2165/00002018-200427080-00010)” has been incorporated.

For disproportionality analyses, references for ROR, PRR, BCPNN, and EBGM have been added, including:

A comparison of measures of disproportionality… (10.1002/pds.66)

Use of proportional reporting ratios… (10.1002/pds.677)

A Bayesian neural network method… (10.1007/s002280050466)

Bayesian Data Mining in Large Frequency Tables…

Quantitative signal detection using spontaneous ADR reporting (10.1002/pds.1742)

These citations now appear in Sections 2.2 and 2.3, ensuring that all analytical tools and terminology systems are properly referenced according to pharmacovigilance methodological standards.

Reviewer 2 – Comment 7

Comment:

The chosen thresholds for disproportionality signals are not fully justified or compared with established best practices. Adjustments for multiple testing are not discussed.

Response:

Thank you for raising this important methodological point. In the revised manuscript, we have strengthened the justification for the disproportionality thresholds and clarified their alignment with established pharmacovigilance best practices. Specifically:

Internationally recognized criteria

We now explicitly state that the thresholds applied in this study—

ROR lower 95% CI > 1,

PRR ≥ 2 with χ² ≥ 4,

IC₀₂₅ > 0,

EB05 > 1

are consistent with recommendations from CIOMS, EMA, WHO Uppsala Monitoring Centre, and widely used in major FAERS/VigiBase analyses.

Multiple disproportionality algorithms

To mitigate false positives related to multiple testing, we utilized four independent algorithms (ROR, PRR, BCPNN, EBGM). A signal was considered meaningful only when multiple methods provided convergent evidence, which is a recognized strategy to improve robustness without overly conservative statistical correction.

Rare events and adjusted thresholds

For very rare events, we clarified that threshold adjustments were applied cautiously and primarily for sensitivity analysis. All such signals were interpreted as exploratory.

Multiple testing

We added a statement noting that formal multiplicity corrections (e.g., Bonferroni) are not standard in spontaneous reporting system analyses due to their potential to mask important safety signals. Instead, combining multiple algorithms and applying consistent thresholds across SOCs and PTs serves as a practical balance between sensitivity and specificity.

These clarifications have been incorporated into Section 2.3 and the Limitations section to improve methodological transparency.

Reviewer 2 – Comment 8

Comment:

Without exposure data (number of patients on fondaparinux), the relative risk cannot be estimated. This limitation should be emphasized more.

Response:

We thank the reviewer for this insightful comment. We agree that the absence of drug exposure data in FAERS limits the ability to estimate relative risks and may affect the generalizability of the findings. Accordingly, we have strengthened the second limitation by explicitly stating that the lack of exposure data may restrict the external generalizability of our results across different populations. This revision clarifies the impact of this limitation on the interpretation of disproportionality estimates.

Reviewer 2 – Comment 9

Comment:

Some signals are based on extremely few cases (e.g., abnormal factor X concentration, n=3), yet interpreted as meaningful. Such findings lack statistical power and clinical reliability.

Response:

We thank the reviewer for this important comment. We fully agree that signals based on very few reports—for example, abnormal coagulation factor X concentration (n=3)—lack statistical robustness and clinical reliability. In the revised manuscript, we have softened the interpretation of these findings and now explicitly describe them as exploratory and hypothesis generating rather than clinically conclusive.

We have also clarified that:

the extremely small case counts make the disproportionality estimates inherently unstable; extreme ROR/PRR values may reflect statistical fluctuation rather than true drug–event associations; such PTs should be regarded as pharmacovigilance alerts rather than confirmed adverse reactions; further validation is needed using larger datasets or prospective investigations.

These revisions ensure a more cautious and scientifically rigorous interpretation of rare signals.

Reviewer 2 – Comment 10

Comment:

While interesting, stratified results (age, gender, onset) may reflect reporting bias rather than true pharmacological effects. This caveat is insufficiently acknowledged.

Response:

We appreciate the reviewer’s insightful comment. We agree that the stratified findings by age, gender, and time to onset may partly reflect differential reporting behavior rather than true pharmacological differences. In response, we have strengthened the Discussion and Limitations sections to explicitly acknowledge this caveat.

In the revised manuscript, we now:

emphasize that reporting tendencies in FAERS vary across demographic groups and may contribute to observed stratified patterns;

clarify that these subgroup results are exploratory and should not be interpreted as definitive evidence of population specific risk differences;

note that external validation using datasets with detailed clinical context, such as electronic health records, is necessary to confirm these observations.

These revisions ensure a more cautious and scientifically rigorous interpretation of the stratified analyses.

Reviewer 2 – Comment 11

Comment:

The discussion part does not adequately distinguish between well established adverse events (e.g., bleeding, hematoma) and weak, hypothesis generating signals.

Response:

We thank the reviewer for this valuable comment. We fully agree that the Discussion should clearly differentiate between well established adverse reactions (such as bleeding and hematoma) and weak, hypothesis generating signals.

In response, we have revised the Discussion to enhance this distinction:

For well established adverse events consistent with the pharmacological profile of fondaparinux (e.g., bleeding, hematoma, anemia, thrombocytopenia), we explicitly describe them as expected and clinically recognized risks.

For rare signals based on very small numbers of reports (e.g., abnormal factor X concentration, thrombosis with thrombocytopenia syndrome, antiphospholipid syndrome, eosinophilia), we now clearly label these as exploratory and hypothesis generating, noting that they lack statistical robustness and require further confirmation.

We added cautionary phrases (e.g., “should be interpreted with caution,” “exploratory in nature,” “require further validation”) to prevent overinterpretation.

Section 4.3 has been strengthened with explicit discussion of instability due to extremely low case counts.

These revisions ensure a more rigorous and accurate interpretation of both established and exploratory findings.

Reviewer 2 – Comment 12

Comment:

Several figures are descriptive but not critically analyzed. For example, time to onset curves are shown but not contextualized clinically.

response:

We appreciate the reviewer’s helpful comment. We agree that descriptive figures, particularly the time to onset (TTO) curves, should be accompanied by more detailed clinical interpretation. In response, we have expanded the Discussion to contextualize these patterns more clearly.

Specifically, we have:

added a dedicated subsection (Section 4.4) interpreting the TTO curves in light of the pharmacokinetic and pharmacodynamic characteristics of fondaparinux, including its rapid onset of action, approximate 18 hour half-life, and the expected timing of bleeding events;

explained why most events cluster within the first month of treatment and provided possible explanations for delayed cases (e.g., reporting delay, prolonged monitoring, or unrelated clinical factors);

clarified that TTO plots reflect reporting patterns rather than true incidence over time and therefore must be interpreted cautiously;

acknowledged that reporting behavior may influence temporal distributions in spontaneous reporting systems.

These revisions strengthen the clinical relevance of the figures and provide a more comprehensive interpretation of the observed patterns.

Reviewer 2 – Comment 13

Comment:

Captions and legends of figures and tables should be standalone and self-explanatory, revise accordingly.

response:

We thank the reviewer for this helpful suggestion. We agree that figure captions and table legends should be sufficiently detailed and self-explanatory. In response, we have revised all captions and legends to ensure they provide adequate context without requiring reference to the main text.

Revisions include:

explicitly describing the study population, analytical methods, and key metrics within each caption (e.g., “Signal detection based on ROR, PRR, BCPNN, and EBGM”);

defining all abbreviations (SOC, PT, ROR, PRR, EBGM, TTO, etc.) within the corresponding table or figure legend;

adding explanatory notes for graphs involving stratified analyses, temporal trends, and onset distributions;

These revisions ensure that every table and figure is independently interpretable and meets publication standards for clarity.

Reviewer 2 – Comment 14

Comment:

Table formatting is not good for publication, revise for better clarity, readership and impact.

response:

We thank the reviewer for this constructive suggestion. All tables have been thoroughly reformatted to meet international publication standards and to improve clarity and readability.

Specifically, we have:

applied a standardized three line table format, removed unnecessary vertical lines, and ensured consistent alignment across all columns;

revised and clarified column headings, ensuring accuracy and uniform capitalization (e.g., replacing “chisq” with “Chi-square value”);

improved readability by adjusting spacing, column width, and the presentation of long variable names for better visual organization;

ensured formatting consistency across all tables (including Table 1 and Table 2) to create a uniform, professional appearance;

added footnotes defining all abbreviations (PT, SOC, ROR, PRR, etc.) to make each table fully self-explanatory.

These revisions substantially enhance the clarity, presentation quality, and overall impact of the tables.

Reviewer 2 – Comment 15

Comment:

Claims about “new” adverse events would benefit from a systematic side-by-side comparison with FDA/EMA product labelling.

response:

We thank the reviewer for this valuable suggestion. We agree that any statements regarding “new” adverse events should be supported by a systematic comparison with current regulatory product labeling. In response, we have revised the manuscript accordingly.

Specifically:

we conducted a side-by-side comparison of all detected PT-level signals with the most recent FDA prescribing information for fondaparinux (2024 revision);

we clarified that PTs not explicitly described in the label are not interpreted as “new adverse reactions,” but rather as hypothesis generating findings that warrant further evaluation;

wording implying novelty has been removed or softened to avoid over interpretation;

Section 4.3 has been updated to explicitly emphasize that these rare signals are exploratory and require confirmation in future studies.

These revisions ensure that the manuscript maintains an appropriately cautious tone and aligns with regulatory and pharmacovigilance standards.

Reviewer 2 – Comment 16

Comment:

Statements such as recommending monitoring of anti-PF4 antibodies and eosinophils are premature, given the weak evidence base. This is overinterpretation. Revise accordingly.

response:

We thank the reviewer for this important comment. We agree that clinical recommendations—such as monitoring anti-PF4 antibodies or eosinophil levels—are not justified based on rare and weak FAERS derived signals. Such statements may imply a level of evidence that is not supported by spontaneous reporting data. We have therefore revised the manuscript accordingly.

Specifically:

all clinical monitoring recommendations based on rare PT-level findings have been removed;

these events are now consistently described as exploratory or hypothesis generating signals;

we clearly state that FAERS data cannot support diagnostic or management recommendations;

cautionary language was added to emphasize that further epidemiologic or mechanistic studies are needed before drawing any clinical implications.

These revisions ensure a more rigorous and appropriately cautious interpretation of rare immune related findings.

Reviewer 2 – Comment 17

Comment:

The dataset is dominated by US and European cases, with minimal Asian data. This limitation is noted but should be emphasized earlier.

response:

We thank the reviewer for this valuable comment. We agree that the predominance of reports from the United States and Europe, along with the limited representation from Asian regions, may affect the generalizability of our findings. In response, we have further strengthened the corresponding statements in the Limitations section, emphasizing that geographic imbalance may introduce population and practice related biases.

We have also added clarifying remarks in the Discussion to ensure that readers are aware of the potential impact of regional underrepresentation when interpreting the results.

These revisions ensure that this limitation is more clearly acknowledged and appropriately contextualized within the manuscript.

Reviewer 2 – Comment 18

Comment:

The conclusion suggests changes to labeling and practice, but the evidence presented does not justify such strong recommendations.

response:

We appreciate the reviewer’s thoughtful comment. We fully agree that FAERS based disproportionality analyses cannot support strong recommendations regarding product labeling or clinical practice changes. In accordance with this comment, we have revised the Conclusion section and removed any statements that could be interpreted as advocating label updates or clinical management recommendations.

The revised conclusion now:

emphasizes that the findings represent associations rather than causal relationships;

characterizes rare PT-level signals as exploratory and hypothesis generating;

highlights the need for further validation using complementary data sources or prospective studies;

positions the study as contributing to post marketing pharmacovigilance rather than guiding clinical practice.

These revisions ensure the conclusion is appropriately cautious and aligned with the evidentiary limitations of spontaneous reporting data.

Reviewer 2 – Comment 19

Comment:

Several grammatical issues and long sentences reduce readability.

response:

We thank the reviewer for this helpful comment. In response to the noted grammatical issues and long sentence structures, we have undertaken a comprehensive language revision of the manuscript.

Specifically:

long or complex sentences were rewritten or split into shorter, clearer statements;

grammatical errors, including subject–verb agreement and improper preposition use, were corrected;

sentence structures were streamlined to improve clarity while maintaining academic rigor;

overall consistency in terminology and writing style was strengthened across sections;

particular attention was given to the Results, Discussion, and Conclusion sections to improve readability and flow.

These revisions substantially enhance the manuscript’s clarity, readability, and linguistic quality.

Reviewer 2 – Comment 20

Comment:

The terminology “Adverse drug events” vs. “adverse drug reactions” are used interchangeably—should be standardized.

response:

We thank the reviewer for this important comment. We agree that consistent terminology is essential for clarity and scientific accuracy. In response, we have standardized terminology throughout the manuscript.

Specifically:

the manuscript now consistently uses “adverse events (AEs)”, avoiding interchangeable use with “adverse drug reactions (ADRs)” or “adverse drug events (ADEs)”;

all sections—including the Abstract, Methods, Results, Discussion, figures, and tables—were carefully reviewed and revised to ensure complete terminological consistency.

These revisions ensure precision and uniformity in terminology across the manuscript.

Reviewer 2 – Comment 21

Comment:

About 30% of reports lacked outcome data, but this important limitation is buried rather than highlighted.

response:

We thank the reviewer for highlighting this important limitation. We agree that the absence of outcome information in approximately 30% of reports can meaningfully affect the interpretation of clinical severity and outcomes, and therefore deserves greater emphasis.

In response, we have strengthened the wording in Limitation Point 6 by:

explicitly stating that missing outcome data reduce dataset completeness and may compromise accurate assessment of serious adverse events (e.g., hospitalization, death, life- threatening events);

clarifying that this limitation may lead to underestimation of clinical severity or incomplete characterization of event impact;

adding a brief remark in the Discussion noting that outcome missingness is a known constraint of FAERS and must be considered when interpreting results.

These revisions make this limitation more visible and ensure appropriate caution in interpreting the findings.

Reviewer 2 – Comment 22

Comment:

Ethical approval required or provide a statement clarifying that ethical approval was not applicable.

response:

We thank the reviewer for this comment. The FAERS database is a publicly accessible, fully de-identified spontaneous reporting system provided by the FDA. As the dataset contains no protected health information and involves no human subjects, ethical approval is not required for this study.

In accordance with the reviewer’s suggestion, we have added the following statement to the Methods section:

“As FAERS data are de-identified and publicly available, this study did not require institutional ethics approval or informed consent.”

This clarification ensures that the ethical considerations of the study are explicitly addressed.

Reviewer 3 – Comment 1

Comment:

Hematoma/hemorrhage are expected in surgical patients receiving anticoagulants. The FAERS analysis must try to separate background risk (e.g., postoperative bleeding) from drug specific signal.

At minimum, report how many bleeding/hematoma reports included concomitant heparin/LMWH or warfarin or antiplatelet agents.

How many HIT like and TTS reports had recent/concurrent heparin exposure? Provide stratified results.

response:

We thank the reviewer for this important comment. We fully agree that bleeding and hematoma are expected events in surgical patients, and distinguishing background surgical risk from drug specific signals is essential.

In response, we conducted additional analyses and revised the manuscript as follows:

Added stratified analyses for concomitant anticoagulants and antiplatelet agents (Section 3.2.3):

We quantified the proportion of bleeding/hematoma reports involving concomitant heparin/LMWH, warfarin, DOACs, or antiplatelet agents. Key findings include:

Hematoma: 2.6% heparin/LMWH, 3.3% warfarin, 0.9% DOACs, 10.5% antiplatelets;

Hemorrhage: 84.5% with no concomitant anticoagulants;

Muscle hemorrhage: 81.6% with no concomitant anticoagulants.

HIT like and TTS signals—documentation of heparin exposure:

We now report the proportion of HIT like and TTS cases with recent or concurrent heparin exposure:

HIT like: 26.1% had heparin/LMWH exposure;

TTS: case numbers were too small for stable estimation, and we explicitly note this limitation.

Conducted a sensitivity analysis excluding all concomitant antithrombotic therapies (Table 4):

After removing all reports containing UFH, LMWH, warfarin, DOACs, or antiplatelet agents, major bleeding related signals remained significant. This suggests that these associations are not solely attributable to background surgical risk or combined therapy.

Added clarification in the Discussion:

We explain that while FAERS cannot fully separate surgical background risk from drug effects, stratified and exclusion analyses help reduce confounding and improve interpretability.

These revisions address the reviewer’s concerns and strengthen the validity of the reported signals.

Reviewer 3 – Comment 2

Comment:

For the HIT like signals and thrombosis with thrombocytopenia (TTS), present how many reports had recent/concurrent heparin exposure. Without this, attributing HIT to fondaparinux is premature.

response:

We thank the reviewer for this important clarification. We fully agree that, without documenting recent or concurrent heparin exposure, attributing HIT like or thrombosis with thrombocytopenia (TTS) signals to fondaparinux would be premature.

In response, we have revised the manuscript as follows:

Added heparin exposure data for HIT like and TTS reports (Section 3.2.3):

HIT like (n = 115): 26.1% had documented heparin/LMWH exposure.

TTS (n = 4): due to the very small number of cases, a stable estimation was not possible, and this limitation has been explicitly noted.

Expanded the Discussion:

We emphasize that fondaparinux does not bind PF4, and the classic HIT mechanism is not pharmacologically expected.

Rare HIT like reports may relate to prior heparin sensitization, perioperative factors, or immune mediated processes, rather than fondaparinux itself.

These signals are now clearly labeled as exploratory and hypothesis generating, not evidence of causality.

Removed or softened any language implying a causal role of fondaparinux and added cautionary statements to prevent overinterpretation.

These revisions ensure an appropriately cautious interpretation of HIT like and TTS findings and align with established pharmacologic and mechanistic knowledge.

Reviewer 3 – Comment 3

Comment:

While the study identifies specific adverse events, the small number of reports for certain signals (e.g., abnormal concentrations of coagulation factor X) raises concerns regarding the statistical power and clinical significance of these findings.

response:

We thank the reviewer for this important comment. We fully agree that signals based on very small numbers of reports—such as abnormal coagulation factor X concentration (n=3)—lack statistical power and limited clinical interpretability.

Accordingly, we have strengthened the manuscript as follows:

Discussion Section 4.3 has been revised to emphasize that:

extremely small case counts lead to unstable ROR/PRR estimates;

unusually high disproportionality values may reflect statistical fluctuation rather than a true association;

such findings are now clearly characterized as exploratory and hypothesis generating, rather than clinically meaningful adverse reactions.

Limitation Point 4 has been expanded:

We explicitly note that rare signals lack statistical robustness and must be interpreted with caution.

All language implying clinical significance has been softened or removed, and replaced with appropriately cautious expressions such as “exploratory in nature,” “require further validation,” and “should be interpreted with caution.”

These revisions ensure a scientifically rigorous and cautious interpretation of rare, low count signals.

Reviewer 3 – Comment 4

Comment:

The retrospective nature of the analysis does not allow for establishing causality; only associations can be suggested. This aspect significantly limits the implications of the findings in clinical practice.

response:

We thank the reviewer for this important comment. We fully agree that, due to the retrospective and spontaneous nature of FAERS data, causality cannot be established, and only statistical associations can be identified. We have revised the manuscript accordingly to more clearly reflect this limitation.

Specifically:

We added explicit statements in the Abstract, Discussion, and Conclusion indicating that the findings represent disproportionality signals rather than causal relationships.

We adopted more cautious terminology throughout the manuscript, replacing terms such as “induced by” with “associated with,” and referring to findings as “signal detection” or “hypothesis generating.”

The first item in the Limitations section was strengthened, emphasizing that the absence of patient level clinical data, exposure denominators, and a comparator arm makes causal inference impossible.

A cautionary note was added to the Discussion, stating that FAERS based analyses serve as post marketing safety alerts and should not be interpreted as evidence for clinical decision making or label modification.

These revisions ensure that the manuscript accurately reflects the inherent constraints of FAERS data and avoids any implication of causality.

Reviewer 3 – Comment 5

Comment:

Absence of detailed laboratory data (e.g., anti PF4 antibody quantification) diminishes the ability to explore the mechanisms underlying the reported adverse events. Including such data could strengthen the conclusions.

response:

We thank the reviewer for this insightful comment. We fully agree that the absence of detailed laboratory data—such as anti-PF4 antibody levels, coagulation factor activity, or hematologic monitoring—limits the ability to explore potential mechanisms underlying certain adverse events. Because FAERS does not provide patient level laboratory information, mechanistic interpretation is inherently constrained.

In response, we have revised the manuscript as follows:

Added clarification in the Discussion:

We explicitly note that FAERS lacks key laboratory parameters required to investigate mechanisms related to HIT like events, TTS, or immune mediated reactions.

Strengthened the Limitations section:

We state that the absence of laboratory data is a major limitation of spontaneous reporting systems and restricts the ability to draw mechanistic or biological conclusions.

Removed or softened any mechanistic interpretations and replaced them with appropriately cautious language such as “cannot be mechanistically confirmed” and “interpretation is limited by unavailable laboratory data.”

These revisions ensure a more accurate representation of the evidentiary boundaries of FAERS based analyses.

Reviewer 3 – Comment 6

Comment:

The authors should provide a more detailed discussion on the limitations of using FAERS and the implications of biases stemming from spontaneous reporting.

response:

We thank the reviewer for this insightful suggestion. In response, we have expanded the discussion of FAERS related limitations by adding a statement in the first limitation paragraph to explicitly address structural biases inherent to spontaneous reporting systems, including selective reporting and stimulated reporting triggered by regulatory or clinical events. These factors may influence the apparent strength and timing of safety signals and further limit interpretability.

This addition provides a more complete and transparent description of FAERS limitations and aligns the manuscript with standard pharmacovigilance reporting practices.

Comment 8（Reviewer’s comment）

If Excel was used only for tabulation/plotting, clarify how ROR/PRR/BCPNN/EBGM were computed.

response:

We thank the reviewer for this important clarification request. We agree that it is essential to distinguish between software used for visualization and the software used for statistical computation. In response, we have revised the Methods section to clearly specify the analytical workflow:

Excel was used only for tabulation, verification, and figure preparation, and not for calculating any disproportionality metrics.

All signal detection analyses were performed in RStudio, using the following packages:

ROR and PRR: calculated using functions from the epiR and DescTools packages via 2×2 contingency tables;

BCPNN: implemented through the bayesAB package;

EBGM: estimated using the openEBGM package (version 0.8.3).

These details have been added to Section “2.6 Statistical software and tools” to enhance transparency and reproducibility.

These revisions clarify the computational process and ensure methodological rigor consistent with current pharmacovigilance standards.

Reviewer 3 – Comment 9

Comment

Some years/statements may need cited support (for example, the statement about Fondaparinux being contraindicated in the US/Canada for CrCl <30 mL/min — cite the product monograph).

response:

We appreciate the reviewer’s suggestion. In line with this comment, we have revised the statement regarding fondaparinux contraindication in patients with severe renal impairment to refer specifically to the U.S. regulatory context and have added the official FDA prescribing information as supporting evidence.

The revised sentence now reads as follows:

“In the United States, fondaparinux is contraindicated in patients with severe renal impairment (creatinine clearance <30 mL/min) for VTE prevention and treatment (32).”

The corresponding reference has been added to the reference list as:

U.S. Food and Drug Administration. Arixtra (fondaparinux sodium) injection, for subcutaneous use: Prescribing Information. Silver Spring (MD): FDA; 2024. Available from: https://www.accessdata.fda.gov/drugsatfda_docs/label/2024/021345s052lbl.pdf

These revisions provide appropriate documentary support for the regulatory statement and avoid overgeneralization beyond the U.S. setting.

Reviewer 3 – Comment 10

Comment

Compute disproportionality of bleeding/HIT signals for fondaparinux vs enoxaparin, unfractionated heparin, and DOACs. This helps determine whether signals are drug specific or class effects.

Response:

We thank the reviewer for this valuable recommendation. We agree that comparative disproportionality analysis across anticoagulant classes is essential for distinguishing drug specific effects from class wide anticoagulant phenomena. In response, we added a new comparative analysis in the revised manuscript.

Specific updates include:

Comparator drugs added: enoxaparin, unfractionated heparin (UFH), and four DOACs (apixaban, dabigatran, edoxaban, rivaroxaban).

Analytical scope: Disproportionality of bleeding related PTs (hematoma, haemorrhage) and HIT/HIT like events was computed using all four algorithms (ROR, PRR, BCPNN, EBGM).

Key findings:

Bleeding and hematoma signals were consistently elevated across all anticoagulants, indicating a class effect.

HIT related disproportionality occurred only with UFH and fondaparinux, whereas DOACs showed no signal, suggesting potential mechanistic specificity.

Placement: The comparative findings are presented in Section 3.2.3, with full details in Supplementary Table 3.

These additions enhance the scientific rigor and help clarify whether observed safety signals reflect drug specific or class wide effects.

Reviewer 3 – Comment 11

Comment

Define all abbreviations on first use (e.g., AE, PT, SOC, TTO, HIT, VITT).

Response:

We thank the reviewer for this helpful suggestion. In accordance with the comment, we carefully reviewed the entire manuscript and ensured that all abbreviations are defined at first use, including AE, PT, SOC, TTO, HIT, and VITT. These revisions improve clarity and consistency throughout the manuscript.

Reviewer 3 – Comment 12

Comment

Most reports are from Europe and the United States, leading to questions about the applicability of findings in other populations, particularly in Asia and other regions with different healthcare practices.

Response:

We appreciate the reviewer’s observation. In the revised manuscript, we expanded the Limitations section to more clearly acknowledge the geographic imbalance of FAERS reports, noting that most cases originate from the United States and Europe, while Asian and other regions remain underrepresented. This imbalance may limit the generalizability of the findings across diverse populations and healthcare systems. These points have been incorporated into the revised Limitations section.

Reviewer 3 – Comment 13

Comment

The authors should consider proposing methods to collect data from underrepresented regions to ensure that findings are applicable globally.

Response:

We thank the reviewer for this valuable and forward looking suggestion. In response, we added a brief section outlining potential approaches to improve representation in future studies, including:

Integrating data from additional pharmacovigilance systems such as EudraVigilance, JADER, and VigiBase;

Conducting multinational real-world studies using electronic health records or insurance databases;

Establishing international collaborative pharmacovigilance networks to improve data availability from underrepresented regions.

These recommendations have been incorporated into the revised Discussion and Limitations sections.

We would like to once again express our sincere gratitude to the Academic Editor and all three reviewers for their thorough evaluation and highly constructive feedback. The comments have greatly strengthened the methodological rigor, clarity of presentation, and clinical relevance of our work. We have carefully addressed each point and revised the manuscript accordingly, with all modifications clearly marked in the revised version.

We believe that the updated manuscript has been substantially improved in terms of scientific robustness, transparency, and readability, and now better aligns with the standards of real- world pharmacovigilance research.

Should further revisions be required, we will be pleased to continue working with the editorial team.

We deeply appreciate the reviewers’ time, expertise, and valuable contributions.
