# Supplementary material for: Disproportionality analysis of fondaparinux associated adverse events based on the FDA adverse event reporting system
Source: PLoS One. 2026 Feb 11;21(2):e0342548. doi: 10.1371/journal.pone.0342548 (PMC12893537; doi:10.1371/journal.pone.0342548)
Supplement: Attachment — Submitted filename: Response_to_Reviewers_auresp_2.docx [file pone.0342548.s010.docx]

Letter to the Editor and Reviewers

Dear Editor and Reviewers,

We sincerely thank the Academic Editor and all reviewers for their careful evaluation of our manuscript entitled“Disproportionality Analysis of Fondaparinux Associated Adverse Events Based on the FDA Adverse Event Reporting System.”We greatly appreciate the constructive comments and helpful suggestions, which have significantly improved the clarity, accuracy, and overall quality of the manuscript.

We have carefully revised the manuscript in accordance with all comments. All changes have been highlighted in the revised version. A point-by-point response is provided below.

**Response to Reviewer #1**

We thank Reviewer #1 for the thoughtful review and helpful suggestions. We have addressed each point in detail as follows:

**Comment 1: Abstract – Define PT with first use.**

Response:

Thank you for this important suggestion. We have revised the Abstract to define Preferred Term (PT) at its first occurrence. Specifically, we now clarify that PT refers to standardized adverse event terminology defined within the Medical Dictionary for Regulatory Activities (MedDRA). This revision improves clarity for readers who may be less familiar with pharmacovigilance terminology.

**Comment 2: In the Results section, the phrase “the age distribution of reporters” appears to refer to reporters rather than patients.**

Response:

We appreciate this careful observation. The wording has been corrected to clarify that the reported age distribution refers to patients, not reporters. The revised sentence now accurately reflects the source and meaning of the demographic data extracted from the FAERS database.

**Comment 3: Fondaparinux should be capitalized when it appears as the first word of a sentence.**

Response:

Thank you for noting this. We have carefully reviewed the entire manuscript and ensured that “Fondaparinux”is capitalized when it appears at the beginning of a sentence, while maintaining lowercase usage elsewhere in accordance with International Nonproprietary Name (INN) conventions.

**Response to Reviewer #2**

Comment:

I have no further suggestions for improvement. The manuscript is now improved and can be considered for publication.

Response:

We sincerely thank Reviewer #2 for the positive evaluation and encouraging comments. We are pleased that the revisions were found to be satisfactory.

**Response to Reviewer #3**

Comment:

The authors have addressed almost all the queries raised by the reviewer. There are none of the queries remaining unanswered.

Response:

We greatly appreciate Reviewer #3’s careful reassessment of the manuscript and the confirmation that all concerns have been adequately addressed.

Once again, we thank the Academic Editor and all reviewers for their valuable time and insightful feedback. We believe that the revisions have substantially strengthened the manuscript, and we hope that the revised version is now suitable for publication.

Sincerely,

Yuan Bian
