# Supplementary material for: Disproportionality analysis of fondaparinux associated adverse events based on the FDA adverse event reporting system
Source: PLoS One. 2026 Feb 11;21(2):e0342548. doi: 10.1371/journal.pone.0342548 (PMC12893537; doi:10.1371/journal.pone.0342548)
Supplement: Attachment — Submitted filename: Reviewer Comments PLOS 40001.docx [file pone.0342548.s006.docx]

The study addresses an important pharmacovigilance question on the safety profile of fondaparinux using FAERS data and multiple disproportionality algorithms (ROR, PRR, BCPNN, EBGM). The dataset is large, spanning 10 years, and the analysis identifies both known and novel safety signals. However, there are significant methodological, interpretational, and presentation issues that limit the scientific rigor and impact.

1. Title of the study should be revised.
2. Introduction is a bit short and avoid redundancy, revise accordingly
3. The manuscript often implies causal links, but FAERS can only generate signals, not establish causality. Stronger disclaimers are needed throughout, revise accordingly for better clarity and impact
4. FAERS reports are prone to underreporting, duplication, missing covariates, and confounding. Although limitations are mentioned, their implications for interpretation are underplayed. Discussion all the issues accordingly throughout the revised manuscript.
5. Cite reference for FAERS in method part.
6. Also cite relevant references in the method part i.e. Medex_UIMA_1.8.3 system, Preferred Terms, (PTs) and System Organ Classes (SOCs) from MedDRA, Reporting Odds Ratio (ROR), Proportional Reporting Ratio (PRR), Bayesian Confidence Propagation Neural Network (BCPNN), and Empirical Bayesian Geometric Mean (EBGM) (20-23)
7. The chosen thresholds for disproportionality signals are not fully justified or compared with established best practices. Adjustments for multiple testing are not discussed.
8. Without exposure data (number of patients on fondaparinux), the relative risk cannot be estimated. This limitation should be emphasized more. This issue may cause negative impact on the generalizability of results.
9. Some signals are based on extremely few cases (e.g., abnormal factor X concentration, n=3 ), yet interpreted as meaningful. Such findings lack statistical power and clinical reliability.
10. While interesting, stratified results (age, gender, onset) may reflect reporting bias rather than true pharmacological effects. This caveat is insufficiently acknowledged.
11. The discussion part does not adequately distinguish between well-established adverse events (e.g., bleeding, hematoma) and weak, hypothesis-generating signals.
12. Several figures are descriptive but not critically analyzed. For example, time-to-onset curves are shown but not contextualized clinically.
13. Captions and legends of figures and tables should be standalone and self-explanatory, revise accordingly.
14. Table formatting is not good for publication, revise for better clarity, readership and impact.
15. Claims about “new” adverse events would benefit from a systematic side-by-side comparison with FDA/EMA product labelling.
16. Statements such as recommending monitoring of anti-PF4 antibodies and eosinophils are premature, given the weak evidence base. This is overinterpretation. Revise accordingly.
17. The dataset is dominated by US and European cases, with minimal Asian data . This limitation is noted but should be emphasized earlier.
18. The conclusion suggests changes to labeling and practice, but the evidence presented does not justify such strong recommendations.
19. Several grammatical issues and long sentences reduce readability.
20. The terminology “Adverse drug events” vs. “adverse drug reactions” are used interchangeably—should be standardized.
21. About 30% of reports lacked outcome data , but this important limitation is buried rather than highlighted.
22. Ethical approval required or provide a statement clarifying that ethical approval was **not applicable**.
